# Systemic angiotensin II does not increase cardiac sympathetic nerve activity in normal conscious sheep

**DOI:** 10.1042/BSR20180513

**Published:** 2018-10-15

**Authors:** Christopher J. Charles, David L. Jardine, Miriam T. Rademaker, A. Mark Richards

**Affiliations:** Christchurch Heart Institute, Department of Medicine, University of Otago, Christchurch, Christchurch, New Zealand

**Keywords:** angiotensins, blood pressure, sympathetic nervous system

## Abstract

While it is well established that centrally injected angiotensin II (Ang II) has potent actions on sympathetic nervous activity (SNA), it is less clear whether peripheral Ang II can immediately stimulate SNA. In particular, the contribution of cardiac sympathetic nerve activity (CSNA) to the acute pressor response is unknown. We therefore examined the effect of incremental doses of intravenous Ang II (3, 6, 12, 24, and 48 ng/kg/min each for 30 min) on CSNA in eight conscious sheep. Ang II infusions progressively increased plasma Ang II up to 50 pmol/l above control levels in dose-dependent fashion (*P*<0.001). This was associated with the expected increases in mean arterial pressure (MAP) above control levels from <10 mmHg at lower doses up to 23 mmHg at the highest dose (*P*<0.001). Heart rate and cardiac output fell progressively with each incremental Ang II infusion achieving significance at higher doses (*P*<0.001)**.** There was no significant change in plasma catecholamines. At no dose did Ang II increase any of the CSNA parameters measured. Rather, CSNA burst frequency (*P*<0.001), burst incidence, (*P*=0.002), and burst area (*P*=0.004) progressively decreased achieving significance during the three highest doses. In conclusion, Ang II infused at physiologically relevant doses increased MAP in association with a reciprocal decrease in CSNA presumably via baroreceptor-mediated pathways. The present study provides no evidence that even low-dose systemic Ang II stimulates sympathetic traffic directed to the heart, in normal conscious sheep.

## Introduction

Sympathetic nervous activity (SNA) is a pivotal element of normal cardiac and circulatory regulation and is enhanced in hypertension, cardiac injury, and heart failure [1]. Many different factors can increase SNA including the renin–angiotensin system, which has the ability to cause both long- and short-term effects [2,3]. While sustained angiotensin II (Ang II) levels in the tissues and the central nervous system (CNS) can increase SNA in the long term, Ang II has not been demonstrated to increase SNA acutely after peripheral injection. Circulating Ang II has direct vasoconstrictor activity, responsible for the so-called ‘acute pressor response’ [4]. The mechanism for this is thought to be direct stimulation of angiotensin I (AT_1_) receptors by Ang II, resulting in vasoconstriction of renal and splanchnic vessels [5]. The rapid increase in blood pressure causes baroreflex-mediated bradycardia and inhibition of renal SNA (RSNA) [6–8]. However, the magnitude of heart rate (HR) slowing is less than expected, and it was suggested that Ang II might also stimulate cardiac SNA (CSNA) centrally [6,7,9]. The most likely mechanism for this was that circulating Ang II (which has a very short plasma half-life) activates receptors in the area postrema which then signal the rostral ventral medulla [10–12]. Consistent with this, CSNA has been demonstrated to progressively increase during 30-min intracerebroventricular infusions of Ang II [13]. To date, however, CSNA has not been recorded during the acute pressor response to circulating Ang II. We therefore aimed to record CSNA continuously during peripheral [iv] Ang II infusions to see if there was a similar increase in CSNA particularly during low dose infusions when baroreflex-induced inhibition was low enough to permit a central effect.

## Experimental

The Animal Ethics Committee of the University of Otago, Christchurch approved the study protocol. Eight Coopworth ewes (Lincoln University Farm, Christchurch, New Zealand) were housed in an air-conditioned, light-controlled room and received a diet of lucerne chaff and food pellets providing 75 mmol of sodium and 150 mmol of potassium per day. A left lateral thoracotomy was performed under general anesthesia (induced by intravenous (IV) diazepam 0.5 mg/kg and ketamine 4 mg/kg, maintained with isoflurane and oxygen) and using approved analgesia (IV carprofen 4 mg/kg; IV buprenorphine 0.005–0.01 mg/kg; regional nerve block with bupivacaine 0.5%/lignocaine 2%) and prophylactic antibiotics (IV cephazolin 20 mg/kg). Up to five stainless steel needle electrodes were inserted and glued in the thoracic cardiac nerves as previously described [14]. Connecting leads were sutured to the mediastinum and exteriorized dorsally through the chest wall. During the same anesthetic, a carotid artery was cannulated (16G Angiocath; Becton Dickinson, Sandy Utah) for the direct measurement of arterial pressure and HR; polyethylene catheters were placed in the jugular veins for blood sampling and measurement of right atrial pressure (RAP), and a Swan-Ganz thermodilution catheter (American Edwards) was placed in the pulmonary artery via the jugular vein for the measurements of cardiac output. The animals were allowed to recover for at least 4 days before experiments.

CSNA recordings were made from pairs of electrodes via a preamplifier with an active probe (World Precision Instruments DAM-80) as previously described [15]. The integrated nerve signal was digitally converted using in-house software (sampling rate 200 Hz), and post-ganglionic efferent sympathetic activity was identified in all animals by the following characteristics: (a) bursts were synchronized to the diastolic phase of the arterial pulse; (b) bursts decreased during sympathetic blockade with hexamethonium infusion (2 mg/kg over 2 h) on day 3 post-thoracotomy; (c) there was an inverse relationship between burst area and diastolic blood pressure during baroreflex tests undertaken on each recording day. Only recordings with a signal-to-noise ratio of greater than two were analyzed. Using these criteria on day 1, the best signal from all possible electrode combinations was selected and used for subsequent recordings. CSNA was quantitated by: (a) counting the number of bursts per minute (burst frequency); (b) counting the number of bursts per 100 heart beats (burst incidence); and (c) measuring the area under the integrated signal per minute (burst area/min) and burst area/100 beats. Sympathetic baroreflex sensitivity may be measured by plotting burst area against diastolic BP; however, the slope is hard to quantitate and the correlation is usually weak [16]. Therefore, threshold variability was estimated by calculating the slope of the regression line between linearly related changes in diastolic BP with CSNA burst incidence [17]. Bursts were counted and then grouped into 2 mmHg BP bins. Slopes were accepted only when there was a linear relationship between six or more consecutive data points. In 90% of sheep, correlation was satisfactory (*R*^2^ > 0.8). To look for horizontal shift of the diastolic BP-CSNA relationship, the *X-*intercepts were calculated for each regression line. Cardiovagal baroreflex sensitivity was measured by calculating the slope of the regression line between linearly related changes in systolic blood pressure and pulse interval over four or more consecutive beats [18]. Slopes were accepted provided pulse interval, and systolic BP changed by more than 5 ms and 1 mmHg, respectively (*R*^2^ > 0.80).

Each animal was studied on two separate occasions receiving IV vehicle control (0.9% saline) and Ang II according to a balanced random order design. Ang II was infused at incremental doses of 3, 6, 12, 24, and 48 ng/kg/min, each for 30 min.

Arterial pressure was recorded using an in-house online data acquisition system commencing 30 min before infusions and continuing for 60 min post-infusions. HR and pressures were digitally integrated in 5 min recording periods and data recorded at preset 15 min intervals throughout the study. Cardiac output (thermodilution) was measured in triplicate (three values within 10%) at preset 15 min intervals for the duration of the study. Venous blood was drawn at preset 30 min intervals during the study protocol. Blood (10 ml total per sample point or 90 ml per study day) was taken into chilled EDTA tubes, centrifuged, and the plasma stored at −80 °C before assay for Ang II [19] and catecholamines [20].

### Statistics

Results are expressed as mean ± S.E.M. Two-way ANOVA with time as a repeated measure was used to determine time and treatment differences between Ang II and control arms of the study. Where significant differences were identified by ANOVA, *a priori* Fisher’s protected least square difference (LSD) tests were used to identify individual time-points significantly different from time-matched control data. Statistical significance was assumed at *P*<0.05.

## Results

**Figure 1:**
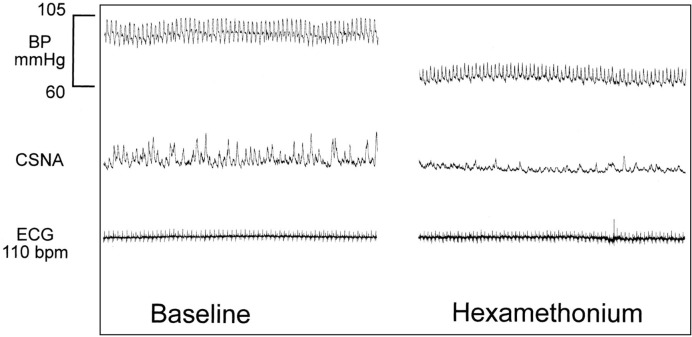
Hexamethonium effect on CSNA. Sample recording of effect of ganglionic blockade with hexamethonium infusion (2 mg/kg IV over 2 h) on integrated CSNA recording.

Experiments were completed without mishap, and data collection was completed. There was no significant difference in baseline measurements of hemodynamic or hormonal variables between the two study days. CSNA burst area is an area under the curve measurement calculated in arbitrary units and as such these data are expressed as percentage change from baseline. Significant baseline differences were observed for CSNA burst frequency (46.1 ± 6.3 versus 55.5 ± 5.7 bursts/min for control and active days respectively, *P*<0.01) and CSNA burst incidence (62.2 ± 7.5 versus 72.1 ± 5.4 bursts/100 beats for control and active days respectively, *P*<0.05). Therefore, these data are also presented as percentage change from baseline. All CSNA recordings were validated as post-ganglionic efferent SNA per methods sections, an example of ganglionic blockade with hexamethonium is shown in Figure 1. In this example, hexamethonium reduced MAP from 92 to 65 mmHg and reduced CSNA burst area from 35 to 6 units/min.

**Figure 2:**
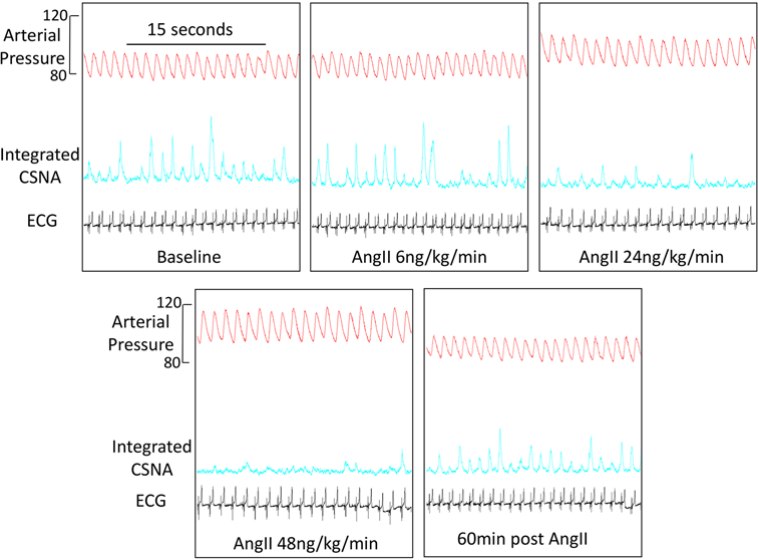
Angiotensin II (AngII) effect on CSNA – sample traces. Sample recordings from a representative sheep of arterial pressure, integrated CSNA, and ECG at baseline after incremental doses of 6, 24, and 48 ng/kg/min Ang II (each infused for 30 min) and then 60 min post-cessation of Ang II infusion (recovery).

Figure 2 shows sample recordings from a representative sheep of arterial pressure, integrated CSNA and ECG at baseline, after three of the incremental doses of Ang II (6, 24, and 48 ng/kg/min) and then 60 min post-cessation of Ang II infusion (recovery). The second dose of Ang II (6 ng/kg/min) induced minimal change in arterial pressure with no activation of CSNA. As arterial pressure progressively increased with higher doses of Ang II, CSNA was progressively reduced. During the post Ang II infusion phase (60 min after cessation of high dose), both arterial pressure and CSNA returned close to baseline levels.

**Figure 3:**
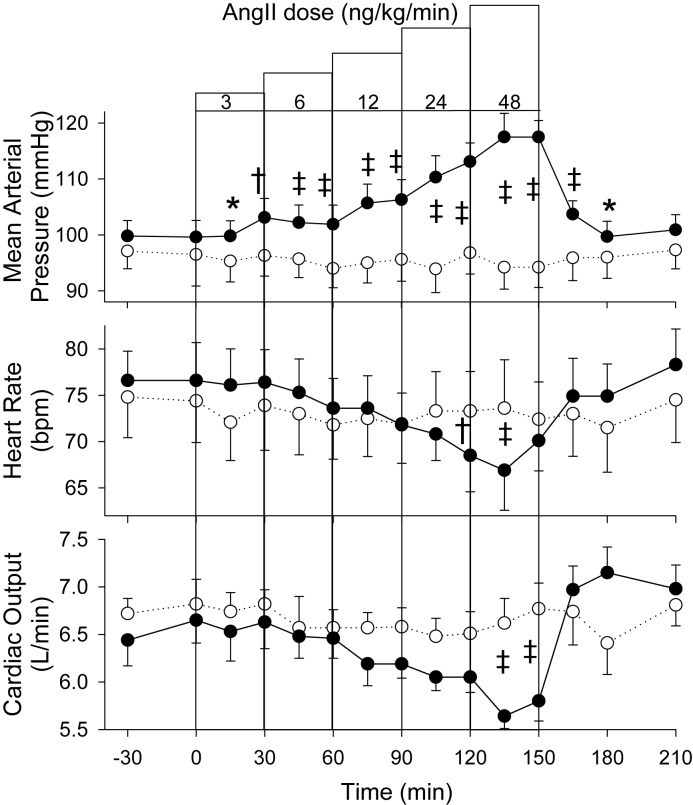
AngII effect on hemodynamics. MAP, HR, and cardiac output responses to incremental dose IV infusions of Ang II (●) or vehicle control (○) in eight sheep. Values shown are mean ± S.E.M. Significant differences were observed for MAP (*P*<0.001), HR (*P*<0.001), and cardiac output (*P*=0.001). Individual time points significantly different from time-matched control (Fisher’s protected LSD from two-way ANOVA) are indicated by *P<0.05, ^†^*P*<0.01, and ^‡^*P*<0.001.

Compared with time-matched control, MAP increased in a dose-dependent fashion in response to increasing doses of Ang II (*P*=0.001) (Figure 3). Increments in MAP were only small during the lowest two doses, being ~7 mmHg above time-matched control levels at 30 and 60 min, but thereafter rose with each dose to eventually be 23 mmHg higher at the end of the highest Ang II dose (117.5 ± 3.0 versus 94.2 ± 3.6 mmHg). HR showed a progressive fall across the Ang II experimental day compared with control (*P*<0.001) with significance only apparent during the top two doses (66.9 ± 4.3 versus 73.6 ± 5.2 at 135 min). Cardiac output showed an overall reduction in response to Ang II (*P*=0.001) with significance evident during the high dose only compared with time-matched control.

**Figure 4:**
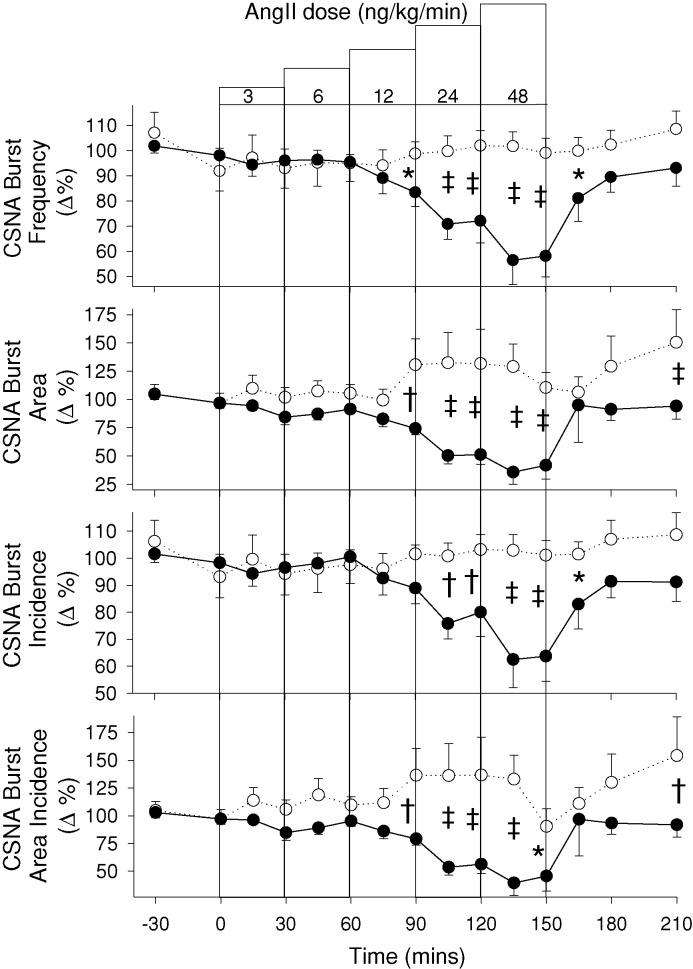
Effect of AngII on CSNA. CSNA responses to incremental dose IV infusions of Ang II (●) or vehicle control (○) in eight sheep. Values shown are mean ± S.E.M. Significant differences were observed for burst frequency (*P*<0.001), burst area (*P*=0.004), burst incidence (*P*=0.002), and burst area incidence (*P*=0.037). Individual time points significantly different from time-matched control (Fisher’s protected LSD from two-way ANOVA) are indicated by *P<0.05, ^†^*P*<0.01, and ^‡^*P*<0.001.

As seen in Figure 4, compared with vehicle control, Ang II induced a fall in all four indices of CSNA; namely, burst frequency (*P*<0.001), burst area (*P*=0.004), burst incidence (*P*=0.002), and burst area/100 beats (*P*=0.037). In all instances, CSNA remained stable during the lowest doses of Ang II and did not begin to fall until the middle dose (12 ng/kg/min) to be significantly reduced by the end of that dose, and falling further during the highest two Ang II doses. At no dose was there any evidence of Ang II-induced activation of CSNA. Baroreflex sensitivity is summarized in Table 1. During the lowest doses of Ang II, CSNA baroreflex sensitivity remained stable at baseline levels: 7.0 ± 2 versus 6.3 ± 1 bursts/100 beats/mmHg (*P*=0.3). During high dose infusion, CSNA baroreflex sensitivity fell marginally to 5.7 bursts/100 beats/mmHg as CSNA decreased, but this change was not significant (*P*=0.08). There was no evidence of a horizontal shift of the baroreflex curve in either direction. Cardiovagal (HR) baroreflex sensitivity did not change from baseline during low or high dose Ang II infusion: 18.1 ± 1.5 ms/mmHg versus 18.0 ± 1.5 (*P*=1.0) and 19.0 ± 1 (*P*=0.6).

**Table 1:** CSNA baroreceptor sensitivity and *X-*intercept in response to vehicle control and incremental doses of 6, 24, and 48 ng/kg/min Ang II each infused for 30 min. Values shown as mean ± S.E.M.

		Baseline	Low dose 6 ng/kg/min Ang II	Medium dose 24 ng/kg/min Ang II	High dose 48 ng/kg/min Ang II
CSNA baroreceptor sensitivity (burst/100 beats/mmHg)	Control	5.79 ± 0.59	5.87 ± 1.20	5.74 ± 0.79	5.57 ± 2.58
	Ang II	6.99 ± 1.02	6.34 ± 1.03	5.65 ± 0.44	5.61 ± 0.68
*X-*intercept	Control	101.7 ± 4.81	102.7 ± 4.22	103.9 ± 3.76	106.0 ± 4.22
	Ang II	109.6 ± 3.58	108.6 ± 3.58	113.1 ± 3.23	113.3 ± 2.28
HR baroreceptor sensitivity (ms/mmHg)	Control	16.4 ± 0.95	17.1 ± 0.85	17.5 ± 1.45	16.5 ± 1.34
	Ang II	18.1 ± 1.59	18.0 ± 1.54	19.0 ± 1.02	17.8 ± 1.43

**Figure 5:**
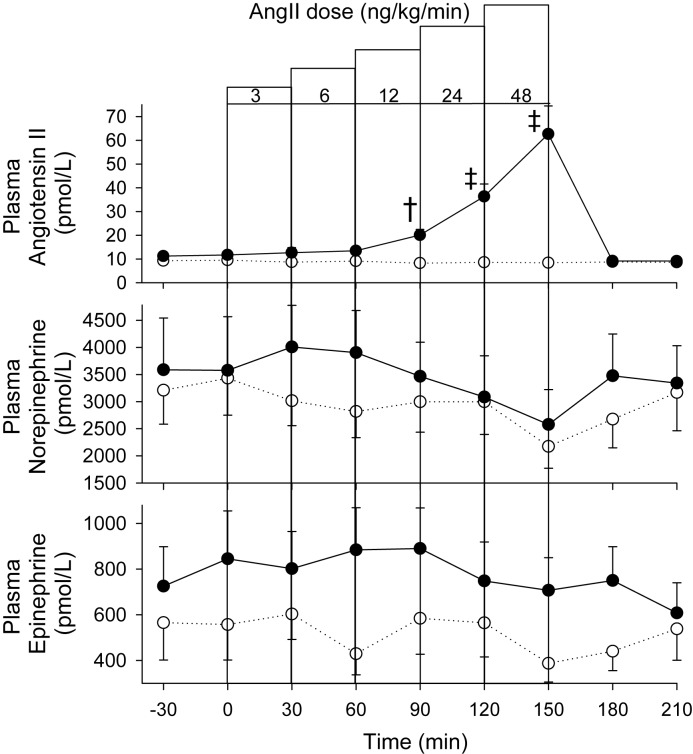
Plasma AngII and catecholamine levels. Plasma Ang II, norepinephrine, and epinephrine response to incremental dose IV infusions of Ang II (●) or vehicle control (○) in eight sheep. Values shown are mean ± S.E.M. Significant differences were observed for Ang II (*P*<0.001). Individual time points significantly different from time-matched control (Fisher’s protected LSD from two-way ANOVA) are indicated by ^†^*P*<0.01 and ^‡^*P*<0.001.

Plasma Ang II levels remained at normal range level (8–10 pmol/l) for the duration of the vehicle control infusions but rose as expected in a dose-dependent fashion during infusion of Ang II (*P*<0.001) to be ~50–55 pmol/l above time-matched control levels by the end of the highest dose (Figure 5). Ang II infusions had no discernable effect on either plasma norepinephrine or epinephrine (Figure 5).

## Discussion

Our results show that IV infusions of Ang II into conscious sheep do not appear to cause any immediate activation of CSNA. Rather, there was a baroreflex-mediated fall in CSNA during the higher doses of Ang II when MAP was clearly increased. Importantly, there was no activation of CSNA during the lower doses of Ang II when the MAP increase was marginal. We observed that as the Ang II infusions incrementally increased MAP, control of CSNA simply moved to the ‘hypertensive end’ of the baroreflex curve. There was no evidence of a right-shift or change in baroreflex gain. These results are consistent with previous studies that have demonstrated the inhibition of SNA to renal, lumbar, splanchnic, and skeletal muscle vessels (with no effects on the sympathetic baroreflex) in response to peripheral Ang II injections [7,8,12,21–23]. Despite the nerve data presented in these studies, there are some previous reports that circulating Ang II has the potential to acutely stimulate SNA at several different levels although most studies support Ang II exerting sympathoexcitatory actions via centrally mediated pathways [23–26]. Reviewing available data together, it appears that increases in circulating Ang II levels have two competing influences on the sympathetic outflow. Ang II acts to increase SNA, although the time course of this action is unclear. On the other hand, Ang II acts indirectly, through increases in arterial pressure caused by its vasoconstrictor action, to stimulate baroreceptors and, thereby, inhibit sympathetic activity. Because infusion of pressor doses of Ang II increases arterial pressure and baroreceptor-evoked sympathoinhibition is quite powerful, sympathoinhibition evoked by Ang II predominates with acute administration of Ang II, thus masking any direct sympathoexcitation [26]. Of note, the acute pressor response to Ang II is associated with less bradycardia and inhibition of SNA than other vasopressor agents [7,12,21]. Authors from these studies suggest that Ang II produces central pressor effects via a hindbrain site, with a selective effect on the arterial baroreflex, and reduces baroreflex inhibition of HR without altering baroreflex inhibition of SNA. Thus, Ang II probably stimulates sympathetic outflow without mediating baroreceptor reflexes. These effects are amplified by barodenervation, and can be decreased by peripheral sympathetic blockade [27,28]. More recent evidence suggests that the CNS is a very important site of Ang II stimulatory action [29]. Delivering very low doses of Ang II more directly to the brain (via the vertebral artery or the fourth ventricle) can mediate rapid increases in BP driven primarily by increased SNA [10]. However, most studies using central injections have demonstrated that although SNA directed to cardiac and splanchnic vessels is increased, RSNA is inhibited [13,30–32]. This is an important example of differential control of the acute sympathetic response [33,34]. Sustained differential activity has also become central to the understanding of sympathetic activity in chronic conditions, namely, heart failure and hypertension [35–38]

The magnitude of the acute pressor response to peripheral Ang II injection is very dependent on its plasma concentration [6,7,10,12]. Therefore, the doses of Ang II infused in the current study were chosen to induce changes in circulating concentrations spanning the spectrum from physiological to clearly elevated levels as seen for the endogenous peptide in our pacing model of moderate-severe heart failure [39]. This resulted in MAP responses ranging from minimal (at the lower doses of Ang II) up to a clear increase (20–25 mmHg). Comparison of MAP with baseline data showed little if any rise in MAP by the end of the second dose of Ang II (101.9 ± 3.4 at 60 min versus 99.6 ± 3.0 mmHg at time 0). There was no evidence of a baroreflex-mediated effect on HR, cardiac output, or CSNA during the low doses of Ang II. By contrast, clear rises in MAP apparent during the higher doses of Ang II (12–48 ng/kg/min) were associated with baroreflex-mediated falls in HR, cardiac output, and CSNA.

The mechanisms involved in the acute pressor response are likely to be different to that observed in animal models of chronic hypertension. In these models, it is well established that circulating Ang II also has the ability to increase blood pressure gradually when given by continuous infusion at doses below the threshold for short-term pressor action [40]. The magnitude and duration of the sympathetic response depend on a number of factors including baroreflex function and background salt status [41–43]. Osborn et al. [35] report that the regional differentiation of the Ang II-salt sympathetic signature is characterized by a transient reduction in RSNA, no change in muscle SNA, and a delayed activation in splanchnic SNA. To date, CSNA levels have not been reported in these models despite evidence that CSNA is disproportionately increased in essential hypertension [44,45]. It is thought that the sustained increase in splanchnic SNA is mediated by the long-term actions of Ang II on central neural pathways.

## Study limitations

The baroreflex results should be tempered by understanding that we only assessed responses to relatively small changes in the linear part of the baroreflex curve [17]. This might explain that why other researchers have documented changes in the cardio-vagal baroreflex curve in response to Ang II. Comparisons with other studies have to be made with caution because the magnitude of the acute pressor response varies depending on animal species, baseline sympathetic activity, use of anesthesia, blood volume status, injection speed, route of injection, and Ang II dose [4,46]. Finally, we did not compare the effects of Ang II on CSNA with other pressor agents that have no central action, therefore cannot completely exclude central stimulation [6].

In conclusion, the current study is the first to examine CSNA response to physiologically relevant systemic infusions of Ang II in a conscious large mammal. Results give no indication that short-term infusions can increase CSNA. Rather, at doses which induce moderate increases in arterial pressure (10–23 mmHg), CSNA is decreased, presumably by baroreflex mechanisms.

## Abbreviations

Ang II: angiotensin II
BP: blood pressure
CNS: central nervous system
CSNA: cardiac sympathetic nerve activity
ECG: electrocardiogram
EDTA: ethylenediaminetetraacetic acid
HR: heart rate
IV: intravenous
LSD: least square difference
MAP: mean arterial pressure
RAP: right atrial pressure
RSNA: renal sympathetic nerve activity
SNA: sympathetic nervous activity
